# Identification of Potential Diuretic and Laxative Drug Candidates from *Avicennia officinalis* L. Bark through *In Vivo* Mice Model Studies and *In Vitro* Gas Chromatography-Mass Spectrometry and Molecular Docking Analysis

**DOI:** 10.1155/2022/4409250

**Published:** 2022-09-19

**Authors:** Md. Nazmul Islam, Md. Fahim Hasan, Aishwarja Dey, Bishwajit Bokshi, Asish Kumar Das, Samir Kumar Sadhu, Nripendra Nath Biswas

**Affiliations:** Pharmacy Discipline, Life Science School, Khulna University, Khulna-9208, Bangladesh

## Abstract

**Background:**

*Avicennia officinalis* is a medicinal plant that has traditionally been used as a diuretic, anti-infective, and antiasthmatic. Our investigation was designed to explore the diuretic and laxative potentials of different fractions of this plant's bark extract as well as the identification of possible drug candidates for the activity.

**Methods:**

Collected bark was extracted in ethanol and fractionated in different polar and nonpolar solvents, i.e., water, chloroform, ethyl acetate, and *n*-hexane. Phytoconstituents were identified following the published protocols and gas chromatography-mass spectrometry (GC-MS). In the diuretic test, Na^+^ and K^+^ ions were measured using a flame photometer whereas the Cl^−^ ion content was measured by titrimetric method against AgNO_3_. In the laxative test, feces amount and consistency were also measured. Molecular docking analysis was conducted using the “Vina Wizard” program in PyRx-Python Prescription 0.8.

**Results:**

Phytochemical analysis indicated that alkaloids, tannins, flavonoids, saponins, glycosides, and terpenoids were detected in the most bioactive crude extracts, whereas alkaloids, terpenoids, saponins, and gums were found in bioactive *n*-hexane fraction and steroids, glycosides, and terpenoids were found positive in chloroform fraction. Almost all the fractions demonstrated a dose-dependent increment of stool production with a soft consistency; however, the chloroform fraction was found to be the most active (*p* < 0.001). The crude extract and *n*-hexane fractions significantly increased (*p* < 0.01) the urinary output at the dose of 200 and 400 mg/kg. The concentrations of Na^+^, K^+^, and Cl^−^ in collected urine were found to be more compared with the control group. The GC-MS analysis identified seven compounds in bioactive *n-*hexane fraction (phenolic and ester-type mainly) whereas seven other compounds (acidic and ester-type mainly) were identified in chloroform fraction. In molecular docking, two drug candidates of this extract (2,4-bis(2-phenylpropan-2-yl)phenol and 2-[4-[2-(dimethylamino)-2-oxo-1,1-diphenylethyl]phenyl]-2-phenylacetic acid) showed excellent binding affinity with the receptor compared with furosemide.

**Conclusion:**

*A. officinalis* bark might be a potential source of bioactive compounds for treating hypertension, edema, and constipation.

## 1. Introduction

Historically, people have been using plants as a precursor of many important traditional as well as modern medicines since ancient times [1]. With the aims of getting novel and better therapeutic effects, reducing the side effects as well as total health care cost, people in this modern age still prefer traditional natural medicines over synthetic drugs in many cases. So far, a wide variety of plants' secondary metabolites (e.g., flavonoids, phenols, glycosides, saponins, stilbenes, tannins, alkaloids, amines, betalains, terpenoids, etc.) have been reported to have many useful pharmacological properties such as anticancer, antibacterial, analgesic, anti-inflammatory, antitumor, and antiviral [2, 3]. The mangrove forest, Sundarbans, which is situated in the southern part of Bangladesh and possesses many plants of diverse ecological adaptive capacity due to high salinity, might be a new source of novel drug lead [4].

The drugs which promote the rate of urine flow are termed diuretics. They mainly do so by increasing the glomerular filtration rate of the kidney and may also change the electrolytes (Na^+^, K^+^, Cl^−^, etc.) composition of the renal tubular fluid. These types of drugs are used in many clinical situations such as edema, hypertension, heart failure, renal obstruction, nephrotic syndrome, ascites, obesity, and pulmonary congestion [5]. Laxative drugs are used to manage constipation which is defined as the hardening of stool; resulting in obstruction in defecation. This uncomfortable public health problem may be alleviated by softening the stool and/or facilitating bowel movement [6]. Plants include a variety of chemicals (terpenoids, flavonoids, alkaloids, and others) that have laxative and diuretic properties [4].

Morphologically, *A. officinalis* is a dense bushy crown-type mangrove tree. The mature ones rise to 15–30 m. The leaves (10 × 5 cm) are shiny green having a rounded apex of a golden-brown bottom side. It blooms small flowers (6 × 10 mm) of orange-yellow to lemon-yellow color. Their smooth barks are dirty green or dark grey in color and do not have flake. Its heart-shaped fruits are green to brown and have a short peak of about 2.5 cm long. It grows scattered or in the colony on the banks of rivers and sometimes near the sea. This species can tolerate high salinity and is mainly found on newly formed mudflats in Bangladesh, Sri Lanka, Thailand, Myanmar, Philippines, Singapore, India, Indonesia, Malaysia, Brunei, etc. [7–9].

Traditionally, this plant is used for treating boils and tumors [10], abscesses, smallpox sores rheumatism, asthma, paralysis, scabies, and snake bites [11]. The bark is used as a diuretic and contraceptive [10]. In earlier research, Ganesh et al. isolated steroid, terpenoid, glycoside, tannin, and resin type important phytoconstituents from the leaves of this plant [12]. Pharmacological reports on leaves and roots include antibacterial [13], anti-inflammatory activity, anticancer activity [14], antioxidant [8], and neuropharmacological properties [15].

Although some attempts were made to prove this plant as an authentic source of traditional medicines, no report could be found on the diuretic and laxative potentials of the bark of this plant. Thus, the present investigation aimed to verify the diuretic and laxative potentials of its barks in a mice model. Additionally, to identify the major compounds responsible for the claimed pharmacological activities, GC-MS analysis and basic pharmacokinetics parameters, for example, absorption, distribution, metabolism, excretion, and toxicity (ADMET) studies of the major compounds (bioactive *n*-hexane and chloroform fraction) were followed by molecular docking analysis with the Na–K–Cl cotransporter-1 (NKCC1), a human protein that aids in the secondary active transport of sodium, potassium, and chloride into cells [16].

## 2. Materials and Methods

### 2.1. Sample Collection and Preparation of Crude Extract

The barks of *A. officinalis* were collected from the Koromjol region, Sundarbans, Bangladesh on May 21, 2017, during the daytime. The plant was identified from National Herbarium, Dhaka, Bangladesh (DACB Accession No.: 46082). The collected barks were dried under shade; ground into a coarse powder with the help of a suitable grinder and 950 g of powder was kept wetting (macerated) in 1000 mL ethanol in a glass container. After 14 days, the contents were filtered and the filtrate was concentrated using a rotary evaporator and a brown-colored powder was obtained. The obtained powder was marked as crude ethanolic extract (yield was 1.47% w/w) and was used for further processing or experiments.

### 2.2. Fractionation of Crude Extract

For solvent-solvent partitioning, 10 g of crude ethanolic extract was taken into a separating funnel and 400 mL of water and an equal amount of *n-*hexane were added and shaken well for proper mixing. The funnel was then kept a while on a ring stand undisturbed for settling the phases distinctly. The nonpolar compounds are more soluble in *n*-hexane than in water. So, they would be dissolved in *n*-hexane. The top (*n*-hexane) and the bottom (water) layers were then collected in separate conical flasks. The water layer was then again transferred to another clean separating funnel and repeated the procedure using chloroform and ethyl acetate solvents. The water fraction was finally dried by the freeze-drying process using a Labocon freeze-dryer (Model: LFD-BT-104). The organic solvents of respective fractions were evaporated using a rotary evaporator and the yields of dried extracts were recorded as 26.00% (for ethyl acetate), 18.67% (for chloroform), 47.33% (for *n*-hexane), and 8.00% (for water). These fractions were further used for animal studies.

### 2.3. Experimental Animals

Both male and female (equal number in a particular test group) young Swiss-albino mice of 7–8 weeks of age and 28–30 g weights were randomly used for all the experiments. The mice were kept in standard environmental conditions at an ambient temperature of 24 ± 1°C, relative humidity of 55–65% with 12 h light:12 h dark cycle in the animal house. All the experiments were conducted in an isolated and noiseless condition following the standard animal ethics guidelines.

### 2.4. Acute Toxicity Test

This test was conducted *in vivo*, according to Lorke's method using crude ethanolic extract [17]. A total of 24 mice were taken and grouped into 4 consisting of 6 in each. Group I, Group II, and Group III were treated with test extracts at different dose levels, i.e., 1, 2, and 3 g/kg body weight, respectively. Group IV was set up as a control group and no treatment was given to them. The animals were monitored for any toxic symptoms or death for the next 2 weeks. On the 14th day, the weight of an individual mouse was measured and recorded.

### 2.5. General Method for the Laxative Test

*In vivo* laxative activity was evaluated following the method of Capasso and his coworkers with minor modifications [18]. A total of 24 mice were allocated into 4 groups consisting of 6 in each. All animals were deprived of food for 12 h before starting the experiment. Group I was administered normal saline (2 mL) orally using a feeding needle and was marked as a control. Similarly, Group II was administered with standard laxative drug bisacodyl in saline (10 mg/kg), Group III and Group IV received the plant extract at the dose of 200 and 400 mg/kg body weight, respectively. After administration, the animals of the individual groups were housed in four different cages where clean filter paper was lined on the bottom for collecting the feces. The excreted feces for up to 16 h periods were collected and weighed. The fecal consistency was also investigated. The experiments were duplicated every time.

### 2.6. General Method for the Diuretic Test

The diuretic test was performed *in vivo* using metabolic cages following the method of Sarker and her coworkers with minor modifications [4]. Briefly, the mice were kept starved for 18 h before starting the experiment. A total of 24 mice were taken and divided into 4 groups consisting of 6 in each. Group I was provided with normal saline (2 mL for each mouse, Vi) orally using a feeding needle. Similarly, Group II was provided with the standard drug furosemide at a dose of 5 mg/kg. Group III and Group IV were provided with plant extracts at a dose of 200 and 400 mg/kg in saline, respectively. Then the animals of the individual groups were placed in four separate metabolic cages for 6 h. During this period, no food or water was given to them. The urine produced by the mice (Vo) was collected every hour in test tubes and was then stored in freezer (0–4°C) for further electrolyte analysis. Further calculation, that is, the urinary excretion was calculated as a ratio of total urinary output (Vo) by total liquid administered (Vi). The diuretic action was calculated as the ratio of urinary excretion in the test group (*U*_ET_) to that of the control group (*U*_EC_). The diuretic activity was calculated as the ratio of diuretic action in the test group (*D*_AT_) and that of the standard group (*D*_AF_). The electrolyte content (Na^+^, K^+^) of the collected urine sample was measured using a flame photometer and Cl^−^ was measured titrimetrically. pH, conductivity, and density were also determined using appropriate apparatus and methods. The experiments were duplicated every time.

### 2.7. Phytochemical Tests for Most Bioactive Fractions

The bioactive ethanolic extract, *n*-hexane, and chloroform fractions of *A. officinalis* bark were subjected to qualitative tests (*in vitro*) for identifying major phytoconstituents using standard protocols and reagents [19, 20].

### 2.8. GC-MS Analysis

The GC-MS analysis was carried out using T Clarus 690 gas chromatography and Clarus SQ 8C mass spectrometer by PerkinElmer at Jashore University of Science and Technology, Bangladesh. One microliter of the extract was injected in splitless mode into the injection port of the GC system. The inlet temperature was set at 250°C, and the oven temperature was programmed as 60°C for 0 min, followed by ramping to 240°C/min at 5°C/min for 4 min. The total run time was 40 min. Helium gas was used as the carrier gas at a constant flow rate of 1.0 mL/min. The interface transfer line temperature was set at 280°C. MS detection was set in scan mode. Quadrupole analyzer temperature was 230°C and ion source temperature was 150°C. Ions were obtained by electron ionization (EI) mode at 70 eV. The scan time and mass ranges were 1 s and 50–600 m/z, respectively. The chemical compounds were identified by comparing the spectral data obtained on the GC-MS with the database of the National Institute Standard and Technology Library [20].

### 2.9. Pharmacokinetic Parameter Study

Basic pharmacokinetic parameters of the compounds found in GC-MS analysis were analyzed with Swiss ADME (http://www.swissadme.ch) [21]. Toxicological information was collected from PubChem (https://pubchem.ncbi.nlm.nih.gov/) [22].

### 2.10. In Silico Molecular Docking Study

#### 2.10.1. Preparation of the Ligands

All the ligands were downloaded from PubChem [22]. The compounds with only 2D structures available were drawn in 3D using Avogadro [23]. After that, all the ligands were optimized using the same program, Avogadro, where the universal force field (UFF) was employed during the process. Finally, ligands were saved in Protein Data Bank (PDB) format.

#### 2.10.2. Preparation of the Protein

The Structure of the human NKCC1 (PDB ID: 6PZT) [24] was obtained from the PDB (http://www.rcsb.org/) [25]. The downloaded protein was cleaned with PyMOL (The PyMOL Molecular Graphics System, Version 2.0 Schrödinger, LLC) and then optimized with the Swiss-PDB viewer [26].

#### 2.10.3. Molecular Docking and Visualization

Molecular docking between the receptor and the ligands was performed using the “Vina Wizard” program in PyRx–Python Prescription 0.8 [27]. The ligands and the receptor were loaded into the program with the proper declaration of the compound, i.e., ligand or macromolecule. Upon completion of the process, the data were obtained along with the docked structures.

Afterward, the docked ligands and the receptor were combined with PyMOL. The combined structures were then visualized with Discovery Studio (BIOVIA, Dassault Systems, Discovery Studio Visualizer, v4.5.0.15071, San Diego: Dassault Systems, ©2005–15). The ligand interactions were observed and snaps were taken of the best poses.

### 2.11. Statistical Analysis

Statistical analysis was performed by Microsoft Excel and Student's unpaired *t*-test (GraphPad Prism software, version 5.0; San Diego, CA, USA). Experimental values were expressed as mean ± standard error of mean (SEM). *p* values < 0.05 were considered to be statistically significant.

## 3. Results

### 3.1. Acute Toxicity

No death or signs of toxicity was observed within 24 h after treatment with the extract; even throughout the whole experimental period of 2 weeks compared with the control group. So, the bark extract was thought to be safe.

### 3.2. Laxative Test

The amount of feces output was found to be more for almost all the test extracts in the experimental animal compared with the control which indicates that the bark has significant laxative activity (Table 1) but the chloroform fraction was found to be the most active. The feces were found to be softer in consistency compared with that of the control.

### 3.3. Diuretic Test

It was found that the ethanolic crude extract and *n*-hexane fraction significantly increased the urinary output at both the tested doses compared with the control. The volume of urine for different test groups and associated computations, i.e., the diuretic action and diuretic activity were performed and shown in Table 2. The comparative ionic content of tested samples is shown in Table 3, where Na^+^ and Cl^−^ concentration increased significantly compared with the control. For predicting the mechanism of diuretic action, different saluretic indices, i.e., natriuretic index, kaluretic index, and carbonic anhydrase inhibition index were calculated using the appropriate formula and have been presented in Table 3. The pH and conductivity were also found to be elevated in test sample urine and presented in Table 4. No demonstrable activity was found in the water fraction and hence not shown in the tables.

### 3.4. Phytochemical Test

The experiments qualitatively indicate the presence of diverse bioactive phytoconstituents in most bioactive three fractions shown in Table 5.

### 3.5. Chemical Composition of *n*-Hexane Fraction by GC-MS Analysis

**Figure 1:**
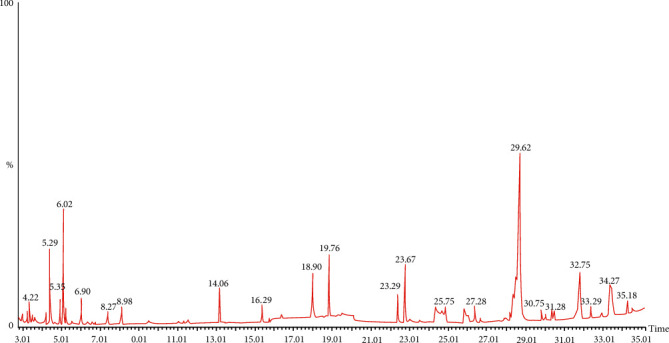
Total ion chromatogram of *n*-hexane fraction of *A. officinalis* bark extract.

The compounds identified in the *n*-hexane fraction by GC-MS analysis were mainly phenolic and ester types. The compound bis(2-ethylhexyl) benzene-1,2-dicarboxylate with a composition of 26.61% was the major constituent. This was followed by phytol with a composition of 5.79% and two other phenolic compounds were identified as phenol, 2,4-bis(2-phenylpropan-2-yl)phenol (5.27%) and 2,4-di-tert-butylphenol (5.07%). The other three major compounds were quantified as 1–2% (Table 6). The peaks shown in the ion chromatogram with a retention time of less than 8.98 min are mainly of some solvents or solvent impurities (Figure 1).

### 3.6. Chemical Composition of Chloroform Fraction by GC-MS Analysis

**Figure 2:**
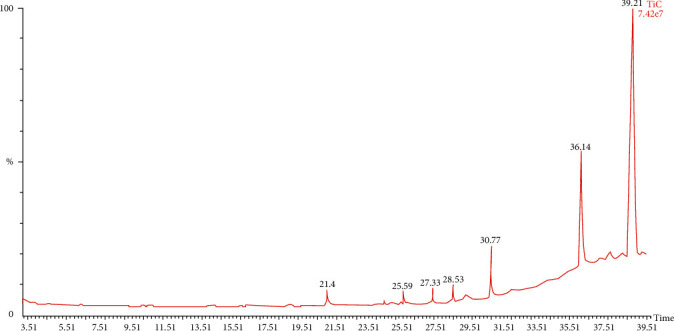
Total ion chromatogram of chloroform fraction of *A. officinalis* bark extract.

**Figure 3:**
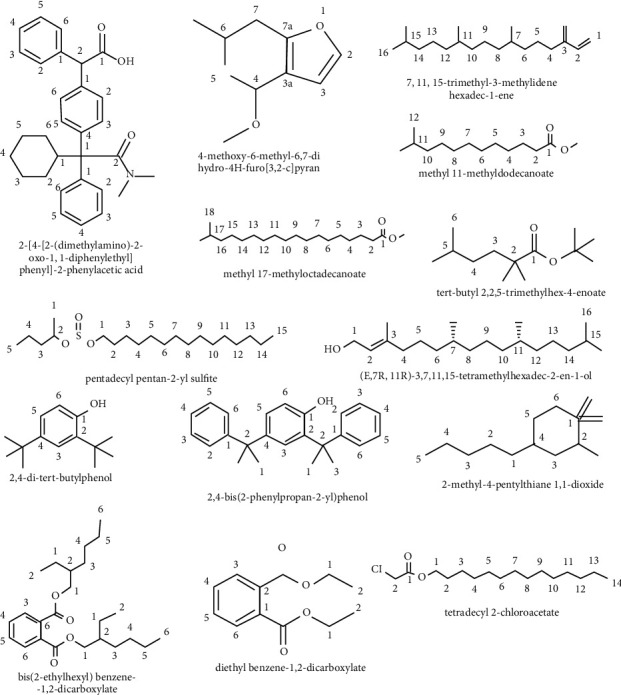
The major compounds of all fractions of the *A. officinalis* bark extract.

The compounds identified in chloroform fraction by GC-MS analysis are of acidic or ester classes. The compound 2-[4-[2-(dimethylamino)-2-oxo-1,1-diphenylethyl]phenyl]-2-phenylacetic acid with a composition of 53.35% was the major constituent. This was followed by tert-butyl 2,2,5-trimethylhex-4-enoate, t-butyl ester with a composition of 20.86%, and two other major compounds were identified as neophytadiene (3.64%) and sulfurous acid, pentadecyl pentan-2-yl sulfite (2.67%). The other three major compounds were quantified as 1–2% (Table 7, Figures 2 and 3).

### 3.7. Pharmacokinetic Parameter Analysis

The compounds found in the *n*-hexane fraction were reported to be toxic (mild, moderate, or very high) and irritant (mild, moderate, or very high). However, the compounds in the chloroform fraction did not seem to be toxic according to the reports. On the other hand, all of them are druggable according to Lipinski's rule of five. The results are briefly shown in Tables 8 and 9.

### 3.8. In Silico Molecular Docking Analysis

**Figure 4:**
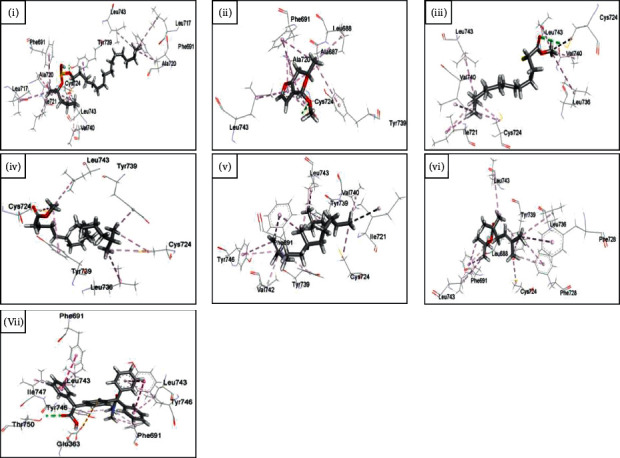
Interaction of human NKCC1 protein with 2-[4-[2-(dimethylamino)-2-oxo-1,1-diphenylethyl]phenyl]-2-phenylacetic acid (i), 4-methoxy-6-methyl-6,7-dihydro-4H-furo[3,2-c]pyran (ii), 7,11,15-trimethyl-3-methylidenehexadec-1-ene (iii), methyl 11-methymethyldodecanoate (iv), methyl 17-methyloctadecanoate (v), pentadecyl pentan-2-yl sulfite (vi), and tert-butyl 2,2,5-trimethylhex-4-enoate (vii).

**Figure 5:**
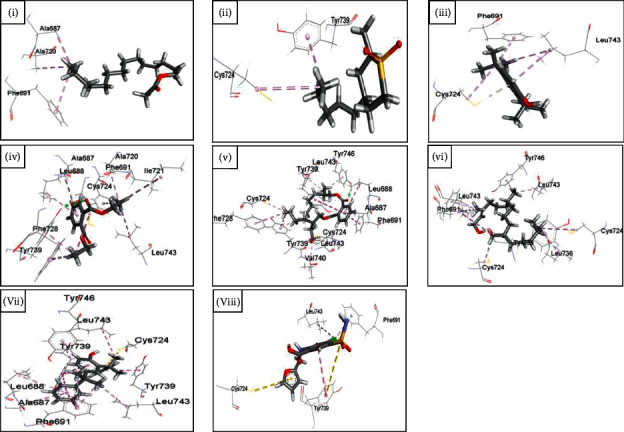
Interaction of human NKCC1 protein with (E,7R,11R)-3,7,11,15-tetramethylhexadec-2-en-1-ol (i), 2,4-bis(2-phenylpropan-2-yl)phenol (ii), 2,4-di-tert-butylphenol (iii), 2-methyl-4-pentylthiane 1,1-dioxide (iv), bis(2-ethylhexyl) benzene-1,2-dicarbdicarboxylate (v), diethyl benzene-1,2-dicarboxylate (vi), tetradecyl 2-chloroacetate (vii), and furosemide (viii).

From the binding affinity and interaction pattern, it can be seen that all the ligands interacted with the receptor analogous to the standard furosemide. Two of them, however, seemed to demonstrate an excellent binding affinity with the receptor compared with furosemide. Those are 2,4-bis(2-phenylpropan-2-yl)phenol (−10.7 kcal/mol) and 2-[4-[2-(dimethylamino)-2-oxo-1,1-diphenylethyl]phenyl]-2-phenylacetic acid (−9.7 kcal/mol). Tables 10 and 11 show the data of docked complexes with the lowest binding affinity implying the best-docked complex for each ligand. Figures 4 and 5 portray the 3D image of the interactions.

## 4. Discussion

Assessments of laxative and diuretic potentials of *A. officinalis* bark extract in different solvent systems in mice models were the main objectives of this research. And hence we prepared the crude extract and then partitioned it into different polar and nonpolar solvents namely water, chloroform, ethyl acetate, and *n*-hexane. Initially, the crude extract was subjected to an acute toxicity test to assess the safety issues of this plant. As no animal died within 24 h after treatment and even no physical abnormalities were observed, that plant part was thought to be safe for further study.

From earlier scientific reports, it has been evident that the extraction method may affect the quantity as well as physiological effects of plant extracts [28], and that was the driving force behind the partition of crude extract in different polar and nonpolar solvents. Liquid-liquid extraction (partitioning) yielded four different fractions with varying polarity where water was more polar (polarity index 10.2) and hexane (0.1) the least. So, the most polar compounds were dissolved in water; medium polar compounds in ethyl acetate (4.4) and chloroform (4.1) fraction; and nonpolar compounds in *n*-hexane, respectively. As the percentage of yield was more in the *n*-hexane fraction (47.33%) than in other fractions, it can be said that the bark might contain mainly nonpolar compounds.

In the laxative test, the fecal output and its consistency in experimental mice were measured in terms of producing laxative effect after treatment with samples. The laxative effect of a standard drug bisacodyl (10 mg/kg) was compared with that of test groups. Notably, almost all the fractions increased the fecal output in mice compared with the control (Table 1). The consistency of feces depends on total water in the large intestinal lumen as well as the water retention capacity of insoluble solids present there. The insoluble solids mainly become available from their diet rich in fibers [29]. From a pharmacological mechanistic action viewpoint, it is evident that the saline and stimulant type laxative drugs exert their actions by modifying either reabsorption or secretion capacity of water in the gut. Some previous investigations on traditional medicines revealed that flavonoid, steroid, alkaloids, saponin, and terpenoid-type plant secondary metabolites produce laxative effects in animals [30–32]. The qualitative phytochemical tests of *A. officinalis* bark performed here also substantiate the existence of such compounds which may produce the claimed laxative effects. Notably, the chloroform fraction was found as the most active here. So, it can be hypothesized that this fraction may contain a higher percentage of terpenoids, saponins, and flavonoids type compounds which may be responsible for its observed bioactivity. Diuretic drugs mainly act by increasing the water excretion rate of the kidney along with the high excretion of electrolytes [33]. A well-known loop diuretic drug furosemide (5 mg/kg dose) was used here as positive control or standard drug. The site of action of this class of drug is ascending loop of Henle where they inhibit Na^+^/K^+^/Cl^−^ symporter [34]. In our study both the positive control and two test samples (ethanolic extract and *n*-hexane fraction) significantly increased the urinary output as well as electrolytes excretion (Tables 2 and 3). The onset of action for furosemide was a bit faster whereas the tested fractions were fairly gradual implying that they may show sustained action. Thus, it is hypothesized that it would reduce the dosing frequency as a diuretic which will certainly increase patient compliance. Previous reports showed that the phytochemicals like terpenoids, flavonoids, and saponins can act as diuretic agents [35]. In this research, phytochemical tests for major bioactive fractions were also conducted which indicated the presence of such constituents (Table 5). And here we hypothesize that the observed diuretic action may be due to their presence which may be synergistically or individually.

As diuretic action is related to electrolytes excretion by urine, we also determined the electrolytes load (Na^+^, K^+^, and Cl^−^) in collected urine. The results revealed that bark extract could effectively modify the electrolytes concentration (Table 3). We thought that conductivity and pH of urine might be other indicative parameters of ionic content and after measurement, they were found in higher amounts compared with the control group (Table 4). Furthermore, some diuretic indices such as natriuretic, kaluretic, and saluretic were calculated to hypothesize the mechanism of such diuretic action and the results supported that the tested samples mechanistically are as like loop diuretic drugs (Table 3). Some earlier scientific reports published that loop diuretics exert their action by modifying natriuresis and kaluresis through inhibition of Na^+^/K^+^/Cl^−^ symporter in the nephron's loop of Henle [5,36]. Clinically, loop diuretics are prescribed for treating or managing patients with salt and water overload (pulmonary edema, cardiac edema, hypertension, etc.) [37]. Moreover, the higher Na^+^ excretion compared with K^+^ is a considerable indication of any good diuretic where potassium ion loss can be minimized [38]. Finally, another important parameter namely carbonic anhydrase (CA) index was also calculated in this study to explore the mechanism of action of these tested extracts. CA index is the ratio of Cl^−^/(Na^+^ + K^+^) excretion and is considered as a substantial indicator of carbonic anhydrase enzyme inhibition and the carbonic anhydrase inhibitory drug class suppresses the activity of that enzyme [39]. These types of drugs are clinically used to treat glaucoma, urinary problem, epilepsy, hypertension, etc. [40]. In our study, the ethanolic extract and *n*-hexane fraction showed good CA inhibitory activity.

GC-MS analysis of plant extract is one of the most powerful tools that are useful for identifying the chemical constituents of plants [41]. The *n*-hexane and chloroform fractions of *A. officinalis* barks were analyzed by GC-MS to detect various compounds. A total of seven major compounds were identified in *n*-hexane (Figures 1 and 3, Table 6) and seven compounds were identified in chloroform fraction (Figures 2 and 3, Table 7). A recent study conducted by Usman et al. indicated that the component extracted from plant parts depends on the type of solvent [42]. Bis(2-ethylhexyl) benzene-1,2-dicarboxylate (retention time 29.62 min, peak area 26.61%) was identified as the major constituents in nonpolar *n*-hexane fraction while 2-[4-[2-(dimethylamino)-2-oxo-1,1-diphenylethyl]phenyl]-2-phenylacetic acid (retention time 39.21 min, peak area 53.35%) and tert-butyl 2,2,5-trimethylhex-4-enoate (retention time 36.14 min, peak area 20.86%) predominated in polar chloroform fraction (Figure 2, Table 7). The claimed diuretic and laxative activity of the fractions might be attributed to the combined action of the major compounds identified in *A. officinalis* extract.

To further bolster this assumption, ADMET analysis of the major compounds was followed by molecular docking analysis with the NKCC1, a protein that aids in the secondary active transport of sodium, potassium, and chloride ions into cells, as shown in Tables 8 and 9 [16]. This co-transporter protein is a target to be inhibited by loop diuretics like furosemide. The result of molecular docking analysis manifests that almost all of the compounds are good inhibitors of the receptor and the interacting amino acids were analogous to furosemide. The most promising outcome in this context were 2,4-bis(2-phenylpropan-2-yl)phenol and 2-[4-[2-(dimethylamino)-2-oxo-1,1-diphenylethyl]phenyl]-2-phenylacetic acid, which outperformed furosemide by 1.5 times. 2,4-bis(2-phenylpropan-2-yl)phenol, at the same time, is an eye irritant demanding further assessment for its safety in use. On the contrary, 2-[4-[2-(dimethylamino)-2-oxo-1,1-diphenylethyl]phenyl]-2-phenylacetic acid is safe according to its toxicological report making it a preferred candidate as a diuretic agent (Table 11, Figures 4 and 5).

Thus, the result and hypothesis made from this study may suggest that traditional practitioners may use *A. officinalis* barks as a useful and nontoxic natural component to treat or manage the above-mentioned ailments. So, the extracts of *A. officinalis* bark may be exposed as a novel interesting alternative to conventionally used diuretic and laxative drugs.

## 5. Conclusion

Analyzing the above findings, it can be suggested that the bark of *A. officinalis* might be used as complementary/alternative medicine and/or maybe a potential source of new drug discovery targeting constipation, renal complication, obesity, edema, and hypertension. However, further studies are necessary to conclude whether the pharmacological potentialities of *A. officinalis* are due to the synergistic role of their compounds or not. Further, bioassay-guided isolation and identification of bioactive compounds may establish more precise structure-activity relationships in the development of diuretic and laxative drugs from this natural source.

## Figures and Tables

**Table 1:** Effect of crude extract, fractions, and bisacodyl on the weight of feces in mice.

Treatment groups	Weight of feces (g)	Weight of feces (g)	Mean weight of feces (g ± SEM)	% increase in feces weight
	Replication 1	Replication 2		
Control (normal saline 2 mL)	0.56	0.52	0.54 ± 0.03	0
Standard (bisacodyl 10 mg/kg)	0.99	0.95	0.97 ± 0.02	79.74 ± 4.17
Ethanolic extract (200 mg/kg)	0.63	0.68	0.66 ± 0.04^*∗*^	25.92 ± 3.37
Ethanolic extract (400 mg/kg)	1.47	1.38	1.43 ± 0.06^*∗∗*^	174.31 ± 19.7
Fr. *n*-hexane (200 mg/kg)	0.97	0.91	0.94 ± 0.04^*∗∗*^	77.37 ± 1.27
Fr. *n*-hexane (400 mg/kg)	1.04	1.12	1.08 ± 0.06^*∗∗*^	100.38 ± 20.98
Fr. chloroform (200 mg/kg)	1.59	1.43	1.51 ± 0.11^*∗∗*^	187.50 ± 17.68
Fr. chloroform (400 mg/kg)	1.85	1.78	1.82 ± 0.05^*∗∗∗*^	245.69 ± 4.77
Fr. ethyl acetate (200 mg/kg)	0.71	0.63	0.67 ± 0.06	26.31 ± 3.93
Fr. ethyl acetate (400 mg/kg)	1.08	1.21	1.15 ± 0.09^*∗∗*^	116.81 ± 28.91
Fr. wate*r* (200 mg/kg)	1.21	1.34	1.28 ± 0.09^*∗∗*^	133.89 ± 1.70
Fr. water (400 mg/kg)	1.71	1.63	1.67 ± 0.06^*∗∗*^	207.41 ± 30.33

Values are expressed as mean ± SEM, (*n* = 2); ^*∗*^*p* < 0.05, ^*∗∗*^*p* < 0.01, and *p* < 0.001, compared with the control group (Student's unpaired *t*-test).

**Table 2:** Effects of crude extract, fractions, and furosemide on the volume of urine and urinary excretion in mice.

Group	Dose (mg/kg)	Cumulative volume of urine (vo) mL/6 h	Urinary excretion (Vo/Vi) × 100	Diuretic action (*U*_ET_/*U*_EC_)	Diuretic activity (*D*_AT_/*D*_AF_)
Control (normal saline)	2 mL/mice	1.40	11.67 ± 1.18	—	—
Standard (furosemide)	5	6.10	50.84 ± 1.20^*∗∗∗*^	4.36 ± 0.35	—
Ethanolic crude extract	200	5.43	40.84 ± 1.19^*∗∗*^	3.50 ± 0.25	0.80 ± 0.01
Ethanolic crude extract	400	5.94	45.21 ± 0.88^*∗∗*^	3.87 ± 0.32	0.89 ± 0.00
Fr. *n*-hexane	200	2.95	24.59^*∗∗*^ ± 0.59	2.11 ± 0.27	0.48 ± 0.02
Fr. *n*-hexane	400	3.24	26.96^*∗∗*^ ± 1.36	2.31 ± 0.35	0.53 ± 0.04
Fr. chloroform	200	0.51	4.25 ± 0.11	0.36 ± 0.05	0.08 ± 0.01
Fr. chloroform	400	0.67	5.54 ± 0.41	0.47 ± 0.01	0.11 ± 0.00
Fr. ethyl acetate	200	0.89	7.38 ± 0.18	0.63 ± 0.05	0.15 ± 0.01
Fr. ethyl acetate	400	1.95	16.25 ± 0.59	1.39 ± 0.09	0.32 ± 0.00

Values are expressed as mean ± SD (*n* = 2); ^*∗∗*^*p* < 0.01, ^*∗∗∗*^*p* < 0.001 compared with the control group. Vo = total urine output per group, Vi = total liquid administered per group (12 mL), *U*_ET_ *=* urinary excretion in the test group, *U*_EC_ *=* urinary excretion in the control group, *D*_AT_ = diuretic activity in the test group, and *D*_AF_ _*=*_ diuretic activity in the standard group (furosemide).

**Table 3:** Effect of crude extract, most bioactive fractions, and furosemide on electrolytes excretion in urine of treated mice.

Groups	Dose (mg/kg)	Concentrations of ions (mEq/L/6 h)			Saluretic index (electrolyte conc. in test group/electrolyte conc. in control group)			Natriuretic index (Na^+^/K^+^)	Kaluretic index (K^+^/Na^+^)	Carbonic anhydrase index (CAI) [Cl^−^/(Na^+^+K^+^)]
		Na^+^	K^+^	Cl^−^	Na^+^	K^+^	Cl^−^			
Control (normal saline)	2 mL/mice	52.99 ± 3.5	29.06 ± 0.52	65.00 ± 2.50	—	—	—	1.82	0.54	0.79
Standard (furosemide)	5	148.55 ± 9.4^*∗∗∗*^	47.84 ± 1.88^*∗∗∗*^	78.75 ± 3.75^*∗*^	2.80	1.65	1.21	3.11	0.32	0.40
Ethanolic crude extract	200	145.01 ± 6.1^*∗∗∗*^	50.45 ± 1.38^*∗∗∗*^	88.75 ± 1.25^*∗*^	2.74	1.74	1.37	2.88	0.35	0.45
	400	180.40 ± 7.1^*∗∗∗*^	61.41 ± 0.52^*∗∗∗*^	92.50 ± 2.50^*∗*^	3.40	2.11	1.42	2.93	0.34	0.38
Fr. *n*-hexane	200	114.47 ± 3.5^*∗∗∗*^	62.45 ± 1.57^*∗∗∗*^	71.25 ± 3.75^*∗*^	2.67	2.15	1.10	1.83	0.55	0.35
	400	173.32 ± 9.4^*∗∗∗*^	65.06 ± 1.38^*∗∗∗*^	85.00 ± 2.50^*∗*^	3.27	2.24	1.31	2.66	0.38	0.36

Values are expressed as mean ± SEM (*n* = 6); ^*∗*^*p* < 0.05, ^*∗∗*^*p* < 0.01, and ^*∗∗∗*^*p* < 0.001, compared with the control group (Student's unpaired *t*-test).

**Table 4:** Effects of crude extract, fractions, and furosemide on urinary volume, diuretic index, conductivity, pH, and density of urine in mice.

Groups	Dose (mg/kg p.o.)	Urine volume (mL/6 h)	Diuretic index^#^	pH	Conductivity (mS/cm)	Density (g/mL)
Control (normal saline)	2 mL/mice	1.4	—	7.11 ± 0.01	5.23 ± 0.02	0.0591
Standard (furosemide)	5	6.1	4.36	7.09 ± 0.02	14.64 ± 0.47	0.0593
Ethanolic crude extract	200	4.9	3.50	7.09 ± 0.00	16.78 ± 0.47	0.0595
	400	5.43	3.88	7.28 ± 0.01	19.37 ± 0.76	0.0601
Fr. *n*-hexane	200	2.95	2.11	7.49 ± 0.02	14.25 ± 0.02	0.0603
	400	3.24	2.31	7.30 ± 0.03	15.43 ± 0.47	0.0604

Values are expressed as mean ± SEM, (*n* = 6); ^#^diuretic index = urine volume of the test group/urine volume of the control group.

**Table 5:** Compounds found in ethanolic crude extract: its *n*-hexane and chloroform fractions.

Constituents	Ethanolic extract	*n*-Hexane fraction	Chloroform fraction
Reducing sugars	+	−	+
Tannins	+	+	−
Flavonoids	+	−	+
Saponins	+	+	+
Gums	+	+	−
Steroids	+	−	+
Alkaloids	+	+	−
Glycoside	+	−	+
Proteins	+	−	+
Phenol	+	−	−
Terpenoids	+	+	+

*Note.* + indicates presence and − indicates absence. Due to the very poor percentage of yield of the water and ethyl acetate fractions, phytochemical screening could not be performed on those fractions.

**Table 6:** Major compounds in *n*-hexane fraction following GC-MS analysis (Figure 1).

Retention time (min)	% area of peak	Compound's name
8.98	0.85	Tetradecyl 2-chloroacetate
14.08	1.66	2-Methyl-4-pentylthiane 1,1-dioxide
19.76	5.07	2,4-Di-tert-butylphenol
23.67	1.94	Diethyl benzene-1,2-dicarboxylate
29.62	26.61	bis(2-Ethylhexyl) benzene-1,2-dicarboxylate
32.75	5.79	(E,7R,11R)-3,7,11,15-Tetramethylhexadec-2-en-1-ol
34.27	5.27	2,4-bis(2-Phenylpropan-2-yl)phenol

**Table 7:** Major compounds in chloroform fraction following GC-MS analysis (Figure 2).

Retention time (min)	% area of peak	Compound's name
21.04	2.67	Pentadecyl pentan-2-yl sulfite
25.59	0.87	4-Methoxy-6-methyl-6,7-dihydro-4H-furo[3,2-c]pyran
27.33	1.23	Methyl 11-methyldodecanoate
28.53	1.15	Methyl 17-methyloctadecanoate
30.77	3.64	7,11,15-Trimethyl-3-methylidenehexadec-1-ene
36.14	20.86	Tert-butyl 2,2,5-trimethylhex-4-enoate
39.21	53.35	2-[4-[2-(Dimethylamino)-2-oxo-1,1-diphenylethyl]phenyl]-2-phenylacetic acid

**Table 8:** Toxicological profiling of the major compounds found in GC-MS analysis.

Compounds	Molecular weight	Information on toxicity
*n*-Hexane fraction		
Tetradecyl 2-chloroacetate	290.87	—
2-Methyl-4-pentylthiane 1,1-dioxide	218.36	—
2,4-Di-tert-butylphenol	206.32	Orally toxic, causes damage to organs through prolonged or repeated exposure
Diethyl benzene-1,2-dicarboxylate	222.24	Teratogenic, neurotoxic
bis(2-Ethylhexyl) benzene-1,2-dicarboxylate	390.56	May damage fertility; may damage the unborn child
(E,7R,11R)-3,7,11,15-Tetramethylhexadec-2-en-1-ol	296.53	Eye irritant
2,4-bis(2-Phenylpropan-2-yl)phenol	330.46	Eye irritant

Chloroform fraction		
Pentadecyl pentan-2-yl sulfite	362.61	—
4-Methoxy-6-methyl-6,7-dihydro-4H-furo[3,2-c]pyran	168.19	—
Methyl 11-methyldodecanoate	228.37	—
Methyl 17-methyloctadecanoate	312.53	—
7,11,15-Trimethyl-3-methylidenehexadec-1-ene	278.52	—
Tert-butyl 2,2,5-trimethylhex-4-enoate	212.33	—
2-[4-[2-(Dimethylamino)-2-oxo-1,1-diphenylethyl]phenyl]-2-phenylacetic acid	449.54	—

**Table 9:** Pharmacokinetic profiling of the major compounds found in GC-MS analysis.

Molecule	#Rotatable bonds	#H-bond acceptors	#H-bond donors	TPSA (Å^2^)	log P	GI absorption	BBB permeant	Pgp substrate	Lipinski #violations	Bioavailability score	Leadlikeness #violations	Synthetic accessibility
*n*-Hexane fraction												
Tetradecyl 2-chloroacetate	15	2	0	26.3	5.46	High	Yes	No	1	0.55	2	2.91
2-Methyl-4-pentylthiane 1,1-dioxide	4	2	0	42.52	2.94	High	Yes	No	0	0.55	1	3.88
2,4-Di-tert-butylphenol	2	1	1	20.23	3.99	High	Yes	No	0	0.55	2	1.43
Diethyl benzene-1,2-dicarboxylate	6	4	0	52.6	2.3	High	Yes	No	0	0.55	1	1.93
bis(2-Ethylhexyl) benzene-1,2-dicarboxylate	16	4	0	52.6	6.17	High	No	Yes	1	0.55	3	4.12
(E,7R,11R)-3,7,11,15-Tetramethylhexadec-2-en-1-ol	13	1	1	20.23	6.22	Low	No	Yes	1	0.55	2	4.3
2,4-bis(2-Phenylpropan-2-yl)phenol	4	1	1	20.23	5.83	High	No	Yes	1	0.55	1	2.51

Chloroform fraction												
Pentadecyl pentan-2-yl sulfite	19	3	0	54.74	6.59	Low	No	Yes	1	0.55	3	4.81
4-Methoxy-6-methyl-6,7-dihydro-4H-furo[3,2-c]pyran	1	3	0	31.6	1.5	High	Yes	No	0	0.55	1	3.74
Methyl 11-methyldodecanoate	11	2	0	26.3	4.4	High	Yes	No	0	0.55	3	2.2
Methyl 17-methyldodecanoate	17	2	0	26.3	6.54	Low	No	No	1	0.55	2	2.88
7,11,15-Trimethyl-3-methylidenehexadec-1-ene	13	0	0	0	7.07	Low	No	Yes	1	0.55	2	4.08
tert-Butyl 2,2,5-trimethylhex-4-enoate	5	2	0	26.3	3.47	High	Yes	No	0	0.55	2	2.62
2-[4-[2-(Dimethylamino)-2-oxo-1,1-diphenylethyl]phenyl]-2-phenylacetic acid	8	3	1	57.61	4.97	High	Yes	Yes	1	0.85	3	3.37

TPSA: topological polar surface area; Log P: octanol/water partition coefficient; lead-likeness #violations: parameters are 250 ≤ MW ≤ 350; XLOGP ≤ 3.5; #rotatable bonds ≤ 7; synthetic accessibility score: 1 = very easy and 10 = very hard.

**Table 10:** 6PZT grid box dimensions.

	*X*	*Y*	*Z*
Centre	157.520	157.480	165.9229
Dimensions (angstrom)	80.0294	90.7224	75.5527

**Table 11:** Binding affinities with 6PZT.

Ligand	Binding affinity (kcal/mol)	Interacting amino acids	
		Side chain A	Side chain B
*n*-hexane fraction			
Tetradecyl 2-chloroacetate	−5.6	PHE691, ALA720, ALA687	
2-Methyl-4-pentylthiane 1,1-dioxide	−5.6		TYR739, CYS724
2,4-Di-tert-butylphenol	−6.9	LEU743	CYS724, PHE691
Diethyl benzene-1,2-dicarboxylate	−6.2	LEU743	**TYR739**, ALA720, PHE691, ILE721, CYS724, PHE728, ALA687, LEU688
bis(2-ethylhexyl)benzene-1,2-dicarboxylate	−7.7	**TYR746**, CYS724, PHE691, ALA687, LEU688, TYR739, LEU743	VAL740, LEU743, PHE728, CYS724, TYR739
(E,7R,11R)-3,7,11,15-tetramethylhexadec-2-en-1-ol	−6.9	CYS724, LEU743, TYR746, PHE691, LEU736	LEU743, CYS724, TYR739
2,4-bis(2-phenylpropan-2-yl)phenol	−10.7	TYR739, LEU743, TYR746, PHE691, LEU688, ALA687	LEU743, TYR739, CYS724

Chloroform fraction			
Pentadecyl pentan-2-yl sulfite	−6.6	**TYR739**, CYS724, LEU743, ALA720, ILE721, PHE691, LEU717	PHE691, VAL740, LEU743, LEU717, ALA720
4-Methoxy-6-methyl-6,7-dihydro-4H-furo[3,2-c]pyran	−5.5	**CYS724,** ALA720, ALA687, PHE691, LEU688, TYR739	LEU743
Methyl 11-methyldodecanoate	−5.7	LEU743, LEU736, ILE721, VAL740, CYS724	**CYS724**, VAL740, LEU743
Methyl 17-methyloctadecanoate	−6.5	LEU743, CYS724, TYR739	LEU736, TYR739, CYS724
7,11,15-Trimethyl-3-methylidenehexadec-1-ene	−6.7	VAL742, TYR739, ILE721, CYS724, PHE691, TYR746	TYR739, VAL740, LEU743
tert-butyl 2,2,5-trimethylhex-4-enoate	−6	LEU743, PHE728	PHE691, LEU688, CYS724, TYR739, LEU743, LEU736, PHE728
2-[4-[2-(dimethylamino)-2-oxo-1,1-diphenylethyl]phenyl]-2-phenylacetic acid	−9.7	**THR750**, GLU363, TYR746, PHE691, LEU743, ILE747	PHE691, LEU743, TYR746
Furosemide	−6.5	CYS724, LEU743	TYR739, PHE691

Conventional hydrogen bond are indicated in bold.

## Data Availability

The data sets supporting the conclusions of this article are included within the article.
